# Identification of a Novel Melon Transcription Factor CmNAC60 as a Potential Regulator of Leaf Senescence

**DOI:** 10.3390/genes10080584

**Published:** 2019-07-31

**Authors:** Songxiao Cao, Zhenbiao Zhang, Chenghui Wang, Xiaoxu Li, Cun Guo, Liyu Yang, Yongfeng Guo

**Affiliations:** 1 Key Laboratory for Tobacco Gene Resources, Tobacco Research Institute, Chinese Academy of Agricultural Sciences, Qingdao 266101, China; 2 College of Horticulture, Shenyang Agricultural University, Shenyang 110866, China

**Keywords:** NAC transcription factor, leaf senescence, Oriental melon, CmNAC60

## Abstract

NAC transcription factors (TFs) play important roles in plants’ responses to abiotic stresses and developmental processes, including leaf senescence. Oriental melon (*Cucumis melo* var. *makuwa* Makino) is an important vegetable crop in China and eastern Asia countries. However, little is known about the functions of the melon NAC family members. In this study, a phylogenetic tree was constructed to show that CmNAC60 and the senescence regulator AtNAP were in the same cluster, which implied that CmNAC60 might be a NAC related to leaf senescence. The expression analysis of *CmNAC60* in different melon organs showed that the expression of *CmNAC60* was highest in the male flowers and lowest in the hypocotyl. In addition, the expression level of *CmNAC60* in the senescing leaves was significantly higher than in the non-senescing leaves. Similarly, the expression level of *CmNAC60* in the dark-treated leaves was significantly higher than in the untreated leaves. Furthermore, the subcellular localization and transcriptional activation assays indicated that CmNAC60 was a nucleus localized NAC transcription factor with a C-terminal transactivation domain. An analysis of the tissue specific expression showed that the promoter of *CmNAC60* may contain *cis*-acting regulatory elements responsive to leaf senescence. *CmNAC60* overexpressing lines of *Arabidopsis* showed a precocious senescence compared with the wild type (WT). Collectively, our results showed that *CmNAC60* was associated with leaf senescence, and could be potentially utilized in molecular breeding to improve melon yield or to extend the postharvest shelf life by delaying leaf senescence.

## 1. Introduction

As the final phase of plant development, leaf senescence is an essential process for plant growth and development [1]. During leaf senescence, a series of changes at the morphological, physiological, and molecular levels occur, including the loss of chlorophyll; degradation of proteins, nucleic acids, and lipids; and nutrient remobilization [2,3]. Leaf senescence can be regulated by various internal factors, such as age and phytohormones, and environmental factors including abiotic and biotic stresses [1,4]. Upon the onset of senescence, a subset of genes termed as senescence-associated genes (SAGs) are transcriptionally upregulated [3]. In *Arabidopsis*, 6326 genes have been identified as being differentially expressed during leaf senescence [5]. Transcription factors (TFs) are important regulators that can interact with the *cis*-acting elements of the target gene promoters, to activate or repress the transcription of the target genes [6]. A number of genes encoding transcription factors have been identified as *SAGs,* and play regulatory roles in leaf senescence. Several families of TFs, which have important roles in regulating leaf senescence, have been characterized, such as NAC, AP2/ERF, bZIP, WRKY, MYB, and C2H2 zinc-finger [5,7,8,9]. 

NAC TFs are one of the largest plant-specific transcription factor families, and the name NAC is originated from the three genes *no apical meristem* (*NAM*), Arabidopsis *transcription activation factor* (*ATAF*), and *cup-shaped cotyledon* (*CUC*), which contain the conserved DNA-binding domain [10,11]. Typically, a NAC TF contains a highly conserved N-terminal NAC domain, which contains five conserved subdomains (A to E) and a diverse C-terminal domain. The N-terminal NAC domain is responsible for nuclear localization, DNA binding, and the formation of homodimers or heterodimers, while the C-terminal domain can function as a transcriptional activator or repressor [12,13]. The NAC TF family has been identified by genome-wide analysis in many important plant species. To date, 105 NAC genes in *Arabidopsis thaliana* [14], 163 in *populous* [15], 152 in soybean (*Glycine max* L.) [16], 74 in grapvine (*Vitis vinifera*) [17], 180 in apple (*Malus domestica*) [18], 110 in potato (*Solanum tuberosum* L.) [19], and 82 in cucumber (*Cucumis sativus* L.) [20] have been identified. NAC TF family members have been suggested to play an important role in various plant growth and development processes, including cell division [21], aging-induced cell death [22], wood formation [23] and cell wall thickening [24], boundary and shoot meristem formation [25], lateral root formation [26], fruit senescence [27], leaf senescence [28,29,30], programmed cell death [31], and biotic and abiotic stress responses [13,32,33].

Recent studies showed that the NAP subfamily (NAC-like, activated by APETALA 3/PISTILLATA), which are one of the largest subfamilies of NAC TFs, plays important roles in leaf senescence in various plant species [28,34,35,36]. *AtNAP* showed a specific expression during the leaf senescence of *Arabidopsis*. The overexpression of *AtNAP* caused precocious senescence in *Arabidopsis,* and knockout mutants of *AtNAP* displayed a significant delay in leaf senescence [28]. A reduced expression of *OsNAP,* which is the *AtNAP* ortholog in rice, delayed leaf senescence and extended the grain-filling period, with the result of an increase in grain yield [36]. In addition, the over-expression of *GhNAP* could cause precocious senescence in *Arabidopsis* and a down-regulation of *GhNAP* delayed leaf senescence in cotton [34].

Leaf senescence can reduce crop production and is closely related to fruit ripening and postharvest storage, so controlling leaf senescence to regulate the timing of ripening and extend postharvest shelf life could be achieved by the genetic manipulation of senescence regulating genes [2]. For oriental melon, we may improve the yield or extend postharvest shelf life by controlling the genes related to leaf senescence via genetic engineering. 

Recently, the characterization of senescence-related NAC TFs in several plant species has been reported [28,34,35,36,37,38], but little is known about melon NACs. In a previous study, 82 NAC genes have been identified in melon by genome-wide analysis, and *CmNAC60* was reported to be a homologous gene of *Arabidopsis AtNAP* [39]. To study the potential role of *CmNAC60* in leaf senescence, the gene was cloned from oriental melon. In this study, *CmNAC60* was demonstrated to be a nucleus-localized NAC transcription factor with a C-terminal transactivation domain. The expression of *CmNAC60* was significantly higher in the senescing leaves of oriental melon, and the *CmNAC60* overexpression lines of *Arabidopsis* showed precocious senescence.

## 2. Materials and Methods 

### 2.1. Plant Materials and Dark Treatment

The seeds of *Arabidopsis thaliana* ecotype Columbia (Col-0) and transgenic plants were sown in a 1/2 Murashige and Skoog (MS) medium followed by vernalization at 4 °C for 3 days. Then, the seven-day-old seedlings were grown in pots filled with peat soil and vermiculite (1:1, *v*/*v*) in a controlled chamber (16 h light/8 h darkness photocycle; 70%–75% relative humidity; 22 °C). Oriental melon (*Cucumis melo* var. *makuwa* Makino) cultivar “Yumeiren” were grown in pots filled with peat soil and vermiculite (1:1, *v*/*v*) in a greenhouse at 25 ± 2 °C. Tobacco (*Nicotiana benthamiana*) plants for agro-infiltration were grown in a growth chamber (16 h light/8 h darkness photocycle; 70%–75% relative humidity; 25 °C) for four weeks. 

For the dark treatment of *Arabidopsis thaliana*, the tenth leaf from the *Arabidopsis thaliana* plants grown for six weeks was excised and incubated on wet filter paper soaked in 1/2 MS + 3 mM 2-(N-Morpholino) ethanesulfonic acid (MES) (pH = 5.8) in darkness at 22 °C for five days. For the dark treatment of oriental melon, the third leaf of the oriental melon grown for one month was excised and incubated on wet filter paper soaked in 1/2 MS + 3 mM MES (pH = 5.8) in darkness at 22 °C for three and six days.

The tissues harvested from *Arabidopsis thaliana* and oriental melon were immediately frozen in liquid nitrogen and stored at −80 °C until used.

### 2.2. Expression of CmNAC60 in Different Tissues of Oriental Melon 

Different tissues, including seeds, cotyledons, hypocotyls, young leaves, stems, roots, tendrils, female flowers, male flowers, and fruits (15 days after pollination), were used in measuring the tissues-specific expression patterns of *CmNAC60*. In addition, non-senescing and senescing leaves at the same leaf position of oriental melon were used to measure the relative expression level of *CmNAC60*. All of the collected samples were immediately frozen in liquid nitrogen and stored at −80 °C.

### 2.3. Isolation and Bioinformatic Analysis of CmNAC60

The cDNA sequence of *CmNAC60* was obtained from the melon genome database (http://melonomics.net/) [40]. The NCBI accession number of *CmNAC60* is XM_008448163, and the cDNA sequence was amplified by PCR with the CmNAC60-F and CmNAC60-R primers (Supplementary Table S1). Then, the PCR product was purified using a TaKaRa MiniBEST DNA Fragment Purification Kit (Takara, Shiga, Japan), and was cloned into the pMD18-T vector (Takara, Shiga, Japan), followed by transferring into *Escherichia coli* DH5α competent cells (Takara, Shiga, Japan). The clones showing the expected size were confirmed by sequencing.

The theoretical molecular weight and isoelectric point (pI) of CmNAC60 were predicated using ProtParam (https://web.expasy.org/protparam/). The nuclear localization signal of CmNAC60 was predicted using the WoLFPSORT algorithm (http://wolfpsort.org/).

### 2.4. Isolation and Bioinformatic Analysis of the CmNAC60 Promoter

The genomic DNA was extracted from the young leaves of oriental melon using the Plant Genomic DNA Kit (Kangwei Biotech, Beijing, China). The sequence of the *CmNAC60* promoter was obtained from the melon genome database and amplified by PCR with CmNAC60-pro-F and CmNAC60-pro-R primers (Supplementary Table S1). The online search tool PlantCARE (http://bioinformatics.psb.ugent.be/webtools/plantcare/html/) was used to detect putative *cis*-acting regulatory elements in the *CmNAC60* and *AtNAP* promoters.

### 2.5. Multiple Sequence Alignment and Phylogenetic Analysis of CmNAC60

Multiple sequence alignments of the deduced protein sequences of CmNAC60, with other reported NAC protein sequences, were aligned using the ClustalW2 software. Then, the multiple sequence alignment of CmNAC60 with other reported NAC protein sequences related to leaf senescence were displayed with GeneDoc. 

The phylogenetic tree was constructed with the deduced protein sequences of CmNAC60 and other reported NAC protein sequences from different plant species using the MEGA v5.0 software based on the neighbor-joining (NJ) method with 1000 bootstrap replications. The gene names and GenBank accession numbers of the NAC proteins used are as follows: *Arabidopsis thaliana*, AtNAP (NP_564966), AtNAM (AAN15611), ATAF1 (NP_171677), ATAF2 (NP_680161), CUC1 (NP_188135), CUC2 (NP_200206), CUC3 (NP_177768), TIP (AAN72023), ANAC019 (NP_175697), ANAC032 (AEE35979), ANAC047 (NP_187057), ANAC055 (NP_188169), ANAC072 (AEE85335), ANAC092 (NP_198777); *Brassica napus*, BnNAC485 (AAP35056), BnNAC18 (AAP35054), and BnNAC5-8 (AAP35052); *Capsicum annuum*, CaNAC1 (AAW48094); *Cucurbita moschata*, CmNAC1 (AWM96385); *Dendranthema lavandulifolium*, DlNAC1 (ABQ96120); *Glycine max*, GmNAC3 (AAY46123), GmNAC4 (AAY46124), GmNAC6 (AAY46126), GmNAC11 (ACC66315), and GmNAC20 (ACC66314); *Gossypium hirsutum*, GhNAC1 (ACI15341), GhNAC2 (ACI15342), and GhNAC5 (ACI15346); *Hordeum vulgare*, HvNAC6 (CAM57978); *Nicotiana tabacum*, TREN (BAA78417); *Oryza sativa*, OsNAC3 (BAA89797), OsNAC4 (BAA89798), OsNAC5 (BAA89799), OsNAC6 (BAA89800), OsNAC052 (AAT44250), OsNAP (NP_912423), and SNAC1 (ABD52007); *Petunia hybrid*, NAM (CAA63101); *Phaseolus vulgaris*, PvNAP (AAK84884); *Populus euphratica*, PeNAC034 (XP_011003434), PeNAC036 (XP_011029436), and PeNAC045 (XP_011022862); *Solanum lycopersicum*, SINAC1 (ACG50002), SlNAC4 (AGH20612); *Solanum tuberosum*, StNAC (CAC42087); *Tamarix hispida*, ThNAC13 (AFN55273); *Triticum aestivum*, TaNAC2 (AAU08786), TaNAC47 (AMQ48929), TaGRAB1 (CAA09371), TaNAC2D (ADE59447); *Vitis pseudoreticulata,* VpNAC1 (ADC94864); *Vitis vinifera*, VvNAC1 (XP_002282566); *Zea mays*, ZmSNAC1 (AEY78612).

### 2.6. Subcellular Localization of CmNAC60

The full-length coding sequence of *CmNAC60* without a stop codon was amplified by PCR with primers CmNAC60-gfp-in-F and CmNAC60-gfp-in-R (Supplementary Table S1), and inserted into the *Spe*I site of the PYG57 vector under the control of the CaMV 35S promoter, to generate the 35S::*CmNAP*-*GFP* fusion construct using an In-fusion HD Cloning Kit (Takara, Shiga, Japan). The 35S::*CmNAP*-*GFP* fusion protein and the negative control 35S::GFP were separately introduced into *Agrobacterium* strain GV3101, and infiltrated into tobacco (*Nicotiana benthamiana*) leaves via the *Agrobacterium*-mediated transformation method. The transformed *N. benthamiana* leaves were grown under normal conditions for three days, followed by staining with 1 μg/mL 4, 6’-diamidino-2-phenylindole (DAPI) for 30 min. The signals were observed and photographed using confocal laser scanning microscopy (TCS-SP8, Leica, Wetzlar, Germany). 

### 2.7. Transcriptional Activation Assay of CmNAC60

To study the transactivation activity of CmNAC60, the full coding sequence (1-292aa), the N-terminal domain (1-161aa), and the C-terminal domain (161-292aa) were separately cloned into the *EcoR*I site of the pBridge vector by the primers listed in Supplementary Table S1, using an In-fusion HD Cloning Kit (Takara, Shiga, Japan) to fuse with a GAL4 DNA binding domain. Fusion plasmids and the control pBridge vector (negative control) were introduced into the yeast strain *AH109* separately, followed by growing yeasts on SD/-Trp, SD/-His, and SD/-His supplemented with 5-bromo-4-chloro-3-indolyl-a-d-galactopyranoside (X-a-Gal) for four days at 30 °C. The transcriptional activation activities were evaluated based on the growth status of different transformants.

### 2.8. Tissue Specific Expression of CmNAC60 

An 1899 bp DNA fragment upstream of the translational initiation codon of the *CmNAC60* gene was amplified by PCR with primers CmNAC60-pro-in-F and CmNAC60-pro-in-R (Supplementary Table S1), and cloned into the *Hind*III and *Bam*HI sites of the pBI121 vector to construct the P*_CmNAC60_*:*GUS* vector. Then, the construct vector was introduced into *Agrobacterium* strain GV3101, and transformed into *Arabidopsis thaliana* Col-0 by the floral dip method [41]. The positive transgenic plants were obtained by kanamycin (*kan*)resistant screening, and further confirmed by PCR. T_3_ lines were used for further experiments. Seven-day-old plants and different tissues of six-week-old P*_CmNAC60_*:*GUS* transgenic plants were harvest for GUS staining, which was performed as previously described [42]. At least 10 non-senescing and 10 senescing rosette leaves harvested from six-week-old transgenic plants were used for the GUS activity analysis. Different tissues of the P*_CmNAC60_*:*GUS* transgenic plants were imaged with a stereomicroscope (Leica, Germany) or SLR camera (Canon, Japan), followed by bleaching by 75% ethanol.

### 2.9. Generation of Overexpression Transgenic Arabidopsis Plants 

The full-length coding sequence of *CmNAC60* was cloned into the *kpn*I and *Sal*I sites of the pCHF3 vector using an In-fusion HD Cloning Kit (Takara, Shiga, Japan) to generate the 35S::*CmNAC60* vector. The sequences of the primers CmNAC60-in-F and CmNAC60-in-R are listed in Supplementary Table S1. Then, the 35S::*CmNAC60* vector was transformed into *Agrobacterium tumefaciens* strain GV3101, followed by transforming into *Arabidopsis thaliana* Col-0 by the floral dip method. The positive transgenic plants were screened by planting on 1/2 MS + 50 μg/mL *kan* plates, and further confirmed by PCR. T_3_ lines exhibiting *kan* resistance were considered to be homozygous and used for further study.

### 2.10. RNA Isolation, qRT-PCR, and Measurement of Chlorophyll Content

The total RNA from the young leaves of oriental melon and *Arabidopsis thaliana* was extracted using the Ultrapure RNA Kit (Kangwei Biotech, Beijing, China), and reverse transcription was performed using the PrimeScript RT reagent Kit with gDNA Eraser (Takara, Shiga, Japan), according to the manufacturer’s instructions. The qRT-PCR was performed on an ABI 7500 real-time PCR machine with a reaction mixture volume of 20 μL. The *18SrRNA* and *AtActin2* genes were used as the internal standards in oriental melon and *Arabidopsis thaliana*, respectively. *SAG12* and the rubisco small subunit gene (*RBSC*) were used as the leaf senescence marker genes to indicate the degree of leaf senescence of *Arabidopsis thaliana*. The following thermal cycle conditions were used: 95 °C for 30 s, followed by 40 cycles of 95 °C for 5 s, and 60 °C for 34 s. The relative gene expression levels were determined using the 2^-ΔΔCT^ method. All of the sequences of the primers used in the study are shown in Supplementary Table S1. The qRT-PCR reaction results were obtained from three independent replicates.

The chlorophyll was extracted and quantified as described previously [43].

### 2.11. Statistical Analysis 

Each experiment was repeated independently at least three times. Statistical analyses were carried out using SPSS 17.0 software. The data are given as mean ± standard deviation (SD). For one pairwise comparison, the data were compared using Student’s *T*-test for statistical analysis. * *p* < 0.05 was considered statistically significant, and ** *p* < 0.01 was considered extremely significant. For multiple pairwise comparisons, the data were compared using a one-way ANOVA test, and differences were considered statistically significant at *p* < 0.05.

## 3. Results

### 3.1. Sequence Characterization of the CDS and Promoter of CmNAC60

**Figure 1:**
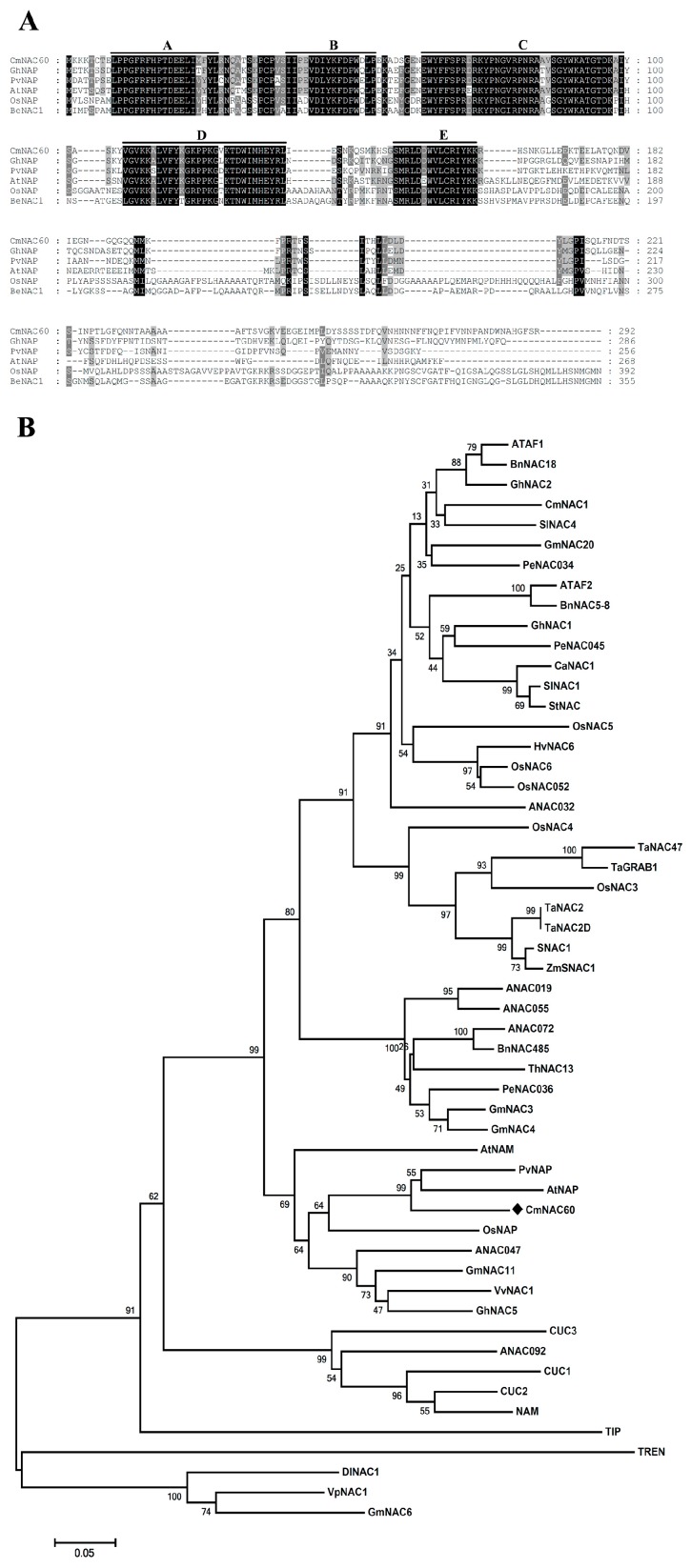
Multiple sequence alignment and phylogenetic analysis of CmNAC60 with other reported NAC protein sequences from different plant species that were functionally characterized. (**A**) Multiple sequences alignment of CmNAC60 with NAC protein sequences regulating leaf senescence from different plant species were aligned using the ClustalW2 software, and displayed with the GeneDoc software. Identical amino acids are shaded in black, and similar amino acids are shaded in gray. The locations of the five highly conserved amino acid motifs (A–E) are marked with overlines. (**B**) The phylogenetic tree was constructed from CmNAC60 with other different NAC proteins using MEGA5.0 with 1000 bootstrap replicates. Bootstrap support values are indicated on each node. CmNAC60 is labeled with a black rhombus.

*CmNAC60* has a length of 879 bp, and was predicted to encode a 292-amino-acid-protein with a molecular weight of 33.32 kD and a pI of 8.25. Furthermore, it was predicted that the deduced CmNAC60 protein was located in the cell nuclei. The multiple sequence alignment of the deduced protein sequence of CmNAC60 with other reported NAC protein sequences related to leaf senescence from different plant species showed that CmNAC60 contained five conserved subdomains (A to E), as indicated by overline (Figure 1A). A phylogenetic tree was constructed with the deduced amino acid sequences of CmNAC60 and other 53 NACs that have been functionally characterized in different plant species. The phylogenetic tree showed that CmNAC60 and AtNAP are in the same cluster (Figure 1B). 

An 1899 bp DNA fragment upstream from the start codon of the *CmNAC60* gene was cloned. Then, two sequence alignments between the nucleotide sequences of *CmNAC60* promoter isolated from oriental melon (*Cucumis melo* var. *makuwa* Makino) in this study, and the *CmNAC60* promoter (named as *GeCmNAC60*) obtained from the melon genome database were done, and 17 single nucleotides were found to be different between the promoters of *CmNAC60* and *GeCmNAC60* (Supplementary Figure S1). Furthermore, the putative *cis*-acting regulatory elements and the locations of these elements in the *CmNAC60* promoter were detected using PlantCARE databases. An analysis of the *CmNAC60* promoter revealed the presence of one *cis*-acting regulatory element, essential for the anaerobic induction, and four *cis*-acting regulatory elements predicted to be responsive to phytohormones, including two ABRE involved in abscisic acid responsiveness, two P-box involved in gibberellin responsiveness, one TCA-element involved in salicylic acid responsiveness, and one TGA-element involved in auxin responsiveness (Supplementary Table S2). The analysis of the *AtNAP* promoter, which has a length of 1961 bp, revealed the presence of one ARE essential for anaerobic induction, one CAT-box related to meristem expression, one GCN4_motif involved in endosperm expression, and four *cis*-acting regulatory elements predicted to be responsive to phytohormones, including five ABRE involved in abscisic acid responsiveness, one TGA-element involved in auxin responsiveness, three CGTCA-motifs, and three TGACG-motifs involved in the MeJA-responsiveness (Supplementary Table S3).

### 3.2. Expression of CmNAC60 in Different Tissues of Oriental Melon

**Figure 2:**
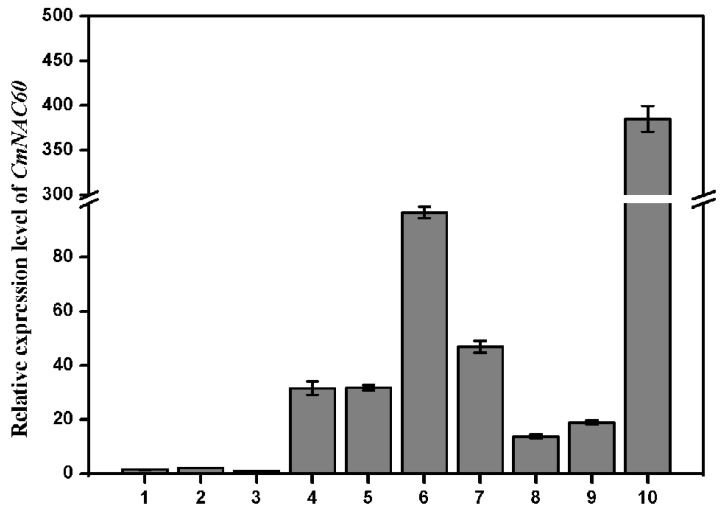
Relative expression levels of *CmNAC60* in different organs of oriental melon. The numbers represent the following: (1) seeds, (2) cotyledon (3) hypocotyls (4) young leaves, (5) stem (6) root (7) tendril (8) 15 d fruit after pollination, (9) female flower, and (10) male flower. The expression levels are calculated relative to the *CmNAC60* expression in the hypocotyls, which was set as 1. The 2^−^^ΔΔ^^CT^ method was used in the qRT-PCR analysis. Data are mean ± standard deviation (SD) from three biological replicates. Bars indicate standard deviation.

RNAs from different tissues of oriental melon were extracted and reverse transcribed into cDNA. Then, qRT-PCR was performed to illustrate the expression of *CmNAC60* in the different tissues of oriental melon. The results revealed that *CmNAC60* had relatively high expression levels in young leaves, stems, roots, and tendrils, and was the highest in male flowers, while the low expression levels were found in seeds, cotyledons, and hypocotyls (Figure 2).

### 3.3. Relative Expression of CmNAC60 during Natural Senescence and Dark Treatment 

**Figure 3:**
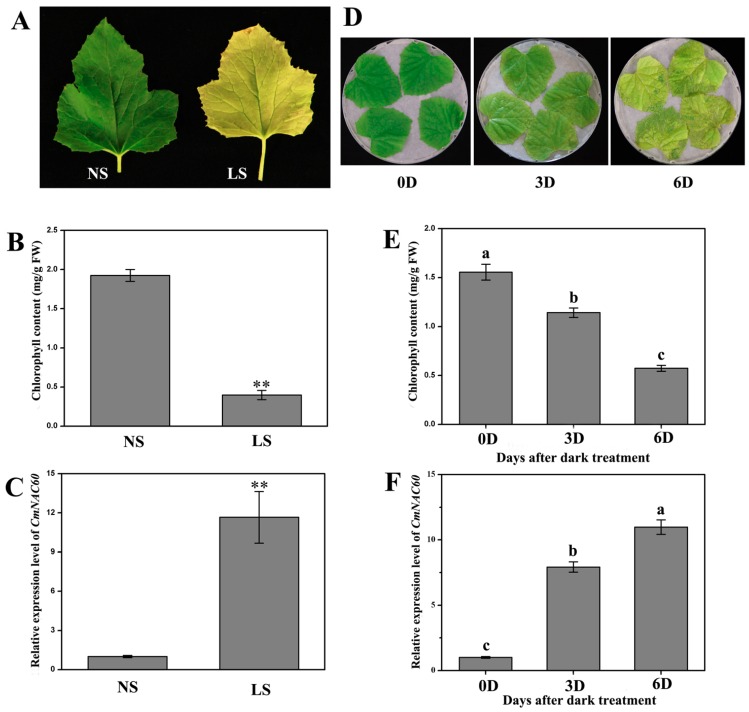
Chlorophyll contents and relative expression levels of *CmNAC60* in oriental melon leaves during natural senescence and dark treatment. (**A**) Oriental melon leaves at non-senescing and naturally senescing stages. NS—a fully expanded and non-senescent leaf; LS—a late senescence leaf. Chlorophyll contents (**B**) and relative expression levels of *CmNAC60* (**C**) at two stages of natural senescence. (**D**) The detached leaves of oriental melon treated in the dark for zero, three, and six days. Chlorophyll contents (**E**) and relative expression levels of *CmNAC60* (**F**) at different dark treatment times. Data are mean ± SD from three biological replicates. The experiments were independently repeated three times with 12 leaves each time. Asterisks indicate statistically significant differences compared with NS (** *p* < 0.01). Different lowercase letters indicate statistically significant differences at *p* < 0.05. Bars indicate standard deviation.

The phylogenetic tree analysis suggested that *CmNAC60* might be related to leaf senescence, so the relative expression levels of *CmNAC60* induced by natural senescence and dark treatment were examined. The relative expression level of *CmNAC60* in the naturally senescing leaves of oriental melon was higher than in the non-senescing leaves (Figure 3C). After dark treatment for three and six days, the relative expression levels of *CmNAC60* in the leaves of oriental melon increased significantly compared with the untreated leaves (Figure 3F). Furthermore, the chlorophyll contents of the melon leaves during natural senescence and dark treatment decreased significantly (Figure 3B,E). 

### 3.4. Subcellular Localization of CmNAC60

**Figure 4:**
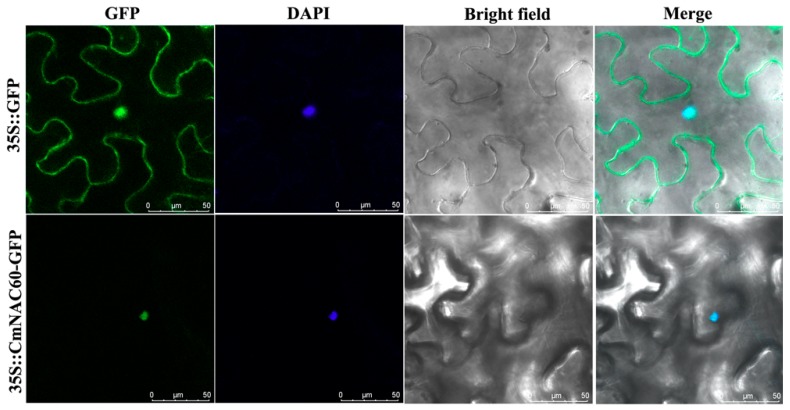
Subcellular localization analysis of CmNAC60 by transient expression in tobacco leaves. Tobacco leaves were infiltrated with *Agrobacterium tumefaciens* carrying the GFP and CmNAC60-GFP fusion constructs, driven by the CaMV35S promoter and detected using a confocal microscope. DAPI staining indicates the position of nucleus. Merge is the overlay images of the GFP fluorescence, DAPI staining, and bright field. The scale bar represents 50 μm.

The subcellular localization of CmNAC60 was determined to find out whether CmNAC60 function as a transcription factor. The full-length coding sequence of *CmNAC60* without a stop codon was fused to the GFP gene sequence in the PYG57 vector. The CmNAC60*-*GFP fusion construct and the GFP control in the PYG57 vector were transiently expressed in the leaves of *N. benthamiana,* respectively, followed by observing the subcellular localization of CmNAC60 using a laser scanning confocal microscope. The results showed that GFP was located to the periphery of the cell, likely in the cytoplasm of the epidermal cells shown in the figure, while the fluorescence signal of the CmNAC60-GFP fusion protein was observed in the cell nucleus, which was confirmed by staining with DAPI (Figure 4).

### 3.5. Transactivation Activity Assay

**Figure 5:**
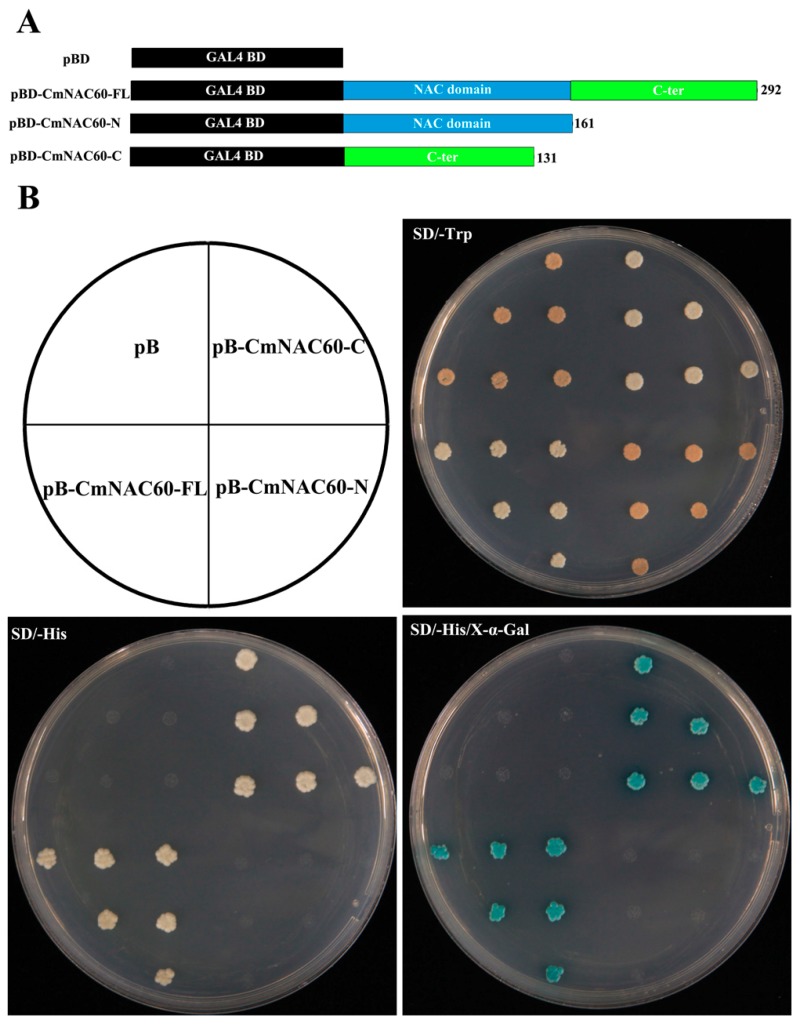
Transactivation activity assay of CmNAC60 in yeast cells. (**A**) Diagram showing that full-length protein (CmNAC60-FL), N-terminal fragment (CmNAC60-N), and C-terminal fragment (CmNAC60-C) of CmNAC60 were fused with a GAL4 DNA binding domain. The pBridge vector was used as a negative control. (**B**) The transformed yeasts were dripped on the SD/-Trp, SD/-His, and SD/-His supplemented with X-α-Gal medium, respectively.

The full-length, the N-terminal, and C-terminal domain coding sequence of CmNAC60 were separately inserted into the pBridge vector, which contains a GAL4 DNA-binding domain, to determine which part of the CmNAC60 protein has the transcriptional activity. These constructs and the empty vector (negative control) were transformed into yeast strain *AH109* cells separately. All of the transformed yeast cells grew well on a SD/-Trp medium, which indicated that the constructs had been successfully transformed into *AH109* cells (Figure 5B).

The yeast cells harboring the full-length coding sequence and the C-terminus of CmNAC60 grew well on the SD/-His medium, while the yeast cells containing the N-terminus of CmNAC60 or the pBridge empty vector did not grow (Figure 5B). Furthermore, the yeast cells harboring the full-length and C-terminus coding sequence of CmNAC60 on the SD/-His medium supplemented with X-a-Gal displayed a blue color, which indicated the activation of the reporter gene *LacZ*. Conversely, the yeast cells containing the N-terminus of CmNAC60 or the pBridge empty vector were not in blue color (Figure 5B). These results showed that the C-terminal part of CmNAC60 has transactivation activities.

### 3.6. Tissue Specific Expression of CmNAC60 

**Figure 6:**
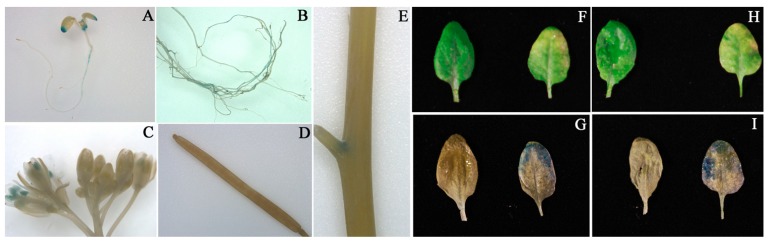
Tissue specific expression of *CmNAC60* in transgenic *Arabidopsis*. (**A**) Seven-day-old seedlings. (**B**) Root, (**C**) inflorescence, (**D**) immature silique, and (**E**) stem of a six-week-old plant. (**F**,**H**) Non-senescing and senescing rosette leaves of a six-week-old plant before GUS staining. (**G**,**I**) Rosette leaves after GUS staining corresponding to (**F**,**H**).

To examine the pattern of the *CmNAC60* expression, the promoter sequence of *CmNAC60* was inserted into the pBI121 vector to construct the P*_CmNAC60_*:*GUS* vector, followed by transferring into *Arabidopsis thaliana* Col-0. In the seven-day-old seedlings, GUS activity was detected in the root and tip regions of the leaf (Figure 6A). In the six-week-old plants, GUS activity was detected in the pistil and stamen of mature flowers and roots, while no GUS activity was detected in immature flowers (Figure 6B,C). In addition, GUS staining was absent in immature siliques, and lower GUS staining was observed in the junction of the stem and the petiole (Figure 6D,E). Furthermore, a significantly elevated GUS activity was observed in the senescing compared with the non-senescing rosette leaves (Figure 6F–I). 

### 3.7. Overexpression of CmNAC60 in Arabidopsis Accelerates Leaf Senescence 

To study the function of *CmNAC60* in the leaf senescence, the transgenic *Arabidopsis* plants overexpressing *CmNAC60* driven by the *CaMV* 35S promoter were generated. Ten T_3_ homozygous transgenic lines were obtained by selection on a medium containing kanamycin, followed by PCR confirmation. Then, two independent homozygous transgenic lines (OE-2 and OE-8 lines) were selected randomly for further study. 

**Figure 7:**
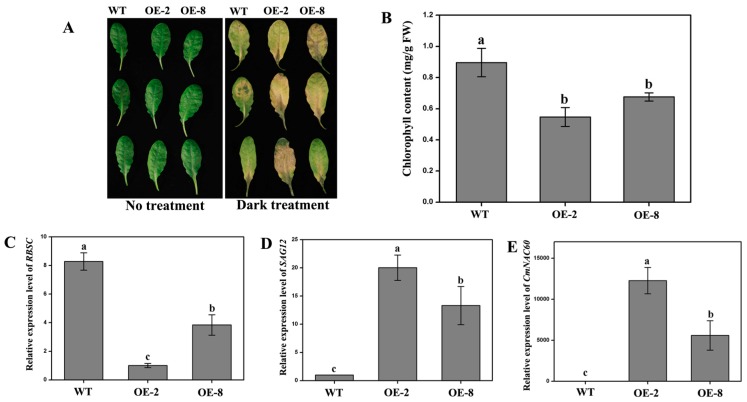
Precocious senescence phenotypes of the detached leaves from *CmNAC60* overexpressing plants after dark-treated for five days. (**A**) Fully expanded rosette leaves of the six-week-old *Arabidopsis* plants were excised and dark-treated for five days. Pictures were taken at zero and five days after dark treatment. (**B**–**E**) Chlorophyll content (**B**), relative expression levels of *RBSC* (**C**)*, SAG12* (**D**), and *CmNAC60* (**E**) in the detached leaves after being dark-treated for five days. The experiments were independently repeated three times, and 12 leaves were used for each replicate. Data are mean ± SD from three biological replicates. Different lowercase letters indicate statistically significant differences at *p* < 0.05. Bars indicate standard deviation.

**Figure 8:**
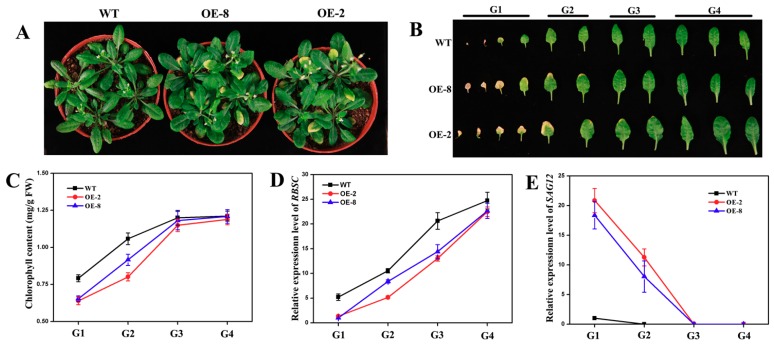
Natural senescence phenotypes of *CmNAC60* overexpressing lines. (**A**,**B**) Phenotypes of *CmNAC60* overexpressing lines OE-2, OE-8, and wild type (WT) under a normal environment after five weeks of growth. (G1–G4) The detached leaves were divided into four groups according to the senescence status. (**C**–**E**) Chlorophyll contents (**C**), the relative expression levels of *RBSC* (**D**), and *SAG12* (**E**) in the four groups of detached leaves corresponding to (**B**). The experiments were independently repeated three times, and 12 plants were used each time. Data are mean ± SD from three biological replicates. Bars indicate standard deviation.

After the detached leaves of *Arabidopsis* were dark-treated for five days, the leaves of the two transgenic lines turned yellow faster than that of the wild type (WT; Figure 7A). In addition, the chlorophyll contents in the leaves of the two transgenic lines decreased significantly compared with the WT (Figure 7B). The relative expression levels of *SAG12* in the leaves of the two transgenic lines increased significantly compared with WT, while *RBSC* decreased markedly (Figure 7C,D). Furthermore, the relative expression levels of *CmNAC60* in the leaves of the two transgenic lines were significantly higher than WT. The relative expression level of *CmNAC60* in the transgenic lines OE-2 was 2.2 times higher than that of OE-8 (Figure 7E). As a result, OE-2 plants showed a more significant early senescence in both the dark-treated (Figure 7) and naturally senescing leaves than the OE-8 plants (Figure 8).

After five-weeks of growth in a growth chamber, the leaves of the two transgenic lines turned yellow faster than the WT (Figure 8A). Then, the detached leaves of the two transgenic lines and WT were divided into four groups (G1–G4; Figure 8B). In the G1 and G2 groups, the chlorophyll contents and relative expression levels of *RBSC* in the transgenic lines were significantly lower than the WT (Figure 8C,D), while the relative expression levels of *SAG12* were higher than the WT (Figure 8E). *SAG12* was used as a leaf senescence marker gene to indicate the degree of leaf senescence of *Arabidopsis thaliana*. The relative expression of the *SAG12* in the dark treatment and natural senescence leaves of the two transgenic lines were higher than the WT, supporting the early senescence phenotypes caused by *CmNAC60* overexpression.

## 4. Discussion

### 4.1. CmNAC60 Is Functionally Related to Leaf Senescence

The NAC TFs are one of the largest plant-specific transcription factor families. Recently, several NAC proteins from different plant species were identified and shown to play different roles in plant growth and development. However, little is known about the functions of the NAC family members in oriental melon. In a previous study, 82 melon NAC genes were identified by a genome-wide investigation, and CmNAC60 was clustered into one group with ANAC029 (AtNAP) in the phylogenetic tree [39].

In this study, the cDNA sequence of *CmNAC60* was obtained from the melon genome database. Multiple sequence alignments of CmNAC60 with other reported NACs that regulate leaf senescence showed that CmNAC60 contained five conserved subdomains. Then, a phylogenetic tree was constructed with the deduced protein sequences of CmNAC60 and 53 other NACs, which were functionally characterized in different plant species, and showed that CmNAC60 and AtNAP were in the same cluster (Figure 1). The results above implied that CmNAC60 may be a NAC functionally related to leaf senescence in oriental melon. 

To further evaluate whether CmNAC60 was related to leaf senescence, the relative expression levels of *CmNAC60* during natural senescence and dark treatment were examined in oriental melon. The relative expression level of *CmNAC60* in natural senescing leaves was increased significantly compared with the non-senescing leaves. Similarly, the relative expression level of *CmNAC60* in the leaves of oriental melon was enhanced by dark treatment (Figure 3). The results above implied that *CmNAC60* may play a role in the leaf senescence of oriental melon.

### 4.2. The *CmNAC60* Promoter May Contain Cis-Acting Regulatory Element Involved in Leaf Senescence

Plant hormones play important regulatory roles in plant senescence [44]. Abscisic acid (ABA), jasmonic acid (JA), and salicylic acid (SA) play positive roles in leaf senescence, while the functions of auxin and gibberellins (GA) on leaf senescence are not clear [1,44,45]. The promoter of *CmNAC60* includes *cis*-acting regulatory elements involved in ABA, SA, auxin, and GA responsiveness, while the promoter of *AtNAP* includes *cis*-acting regulatory elements involved in ABA, auxin, and JA responsiveness. Genes may be involved in leaf senescence via participating hormonal pathways. The promoters of *CmNAC60* and *AtNAP* both contain ABRE, which is involved in abscisic acid responsiveness. *AtNAP, GhNAP,* and *OsNAP* regulate leaf senescence through the ABA pathway [34,36,46]. *CmNAC60* may participate in the ABA signaling pathway in regulating leaf senescence.

To investigate the tissue specific expression of *CmNAC60*, the promoter of *CmNAC60* was cloned, and the P*_CmNAC60_*:*GUS* vector was constructed, followed by transferring into *Arabidopsis thaliana* Col-0. In seven-day-old seedlings, the GUS activity was mainly observed in the tip of the leaves, which represented the oldest, but not yet senescent leaf regions [47]. After the GUS staining of leaves from six-week-old plants, the GUS activity was mainly detected in the senescent parts of the partially senescent leaves, while no GUS activity was detected in the non-senescing leaves (Figure 6). These results above suggested that the *cis*-acting regulatory elements involved in leaf senescence may be present in the promoter of *CmNAC60.*

### 4.3. Relative Expression Difference of CmNAC60 between the Male and Female Flowers of Oriental Melon

The result of the qRT-PCR revealed that the relative expression level of *CmNAC60* was the highest in the male flowers of oriental melon, but was not high in female flowers (Figure 2). The fact that the female flower contains a big ovary might be the cause of the relative expression difference of *CmNAC60* between the male and female flowers. Furthermore, CmNAC60 may have other biological roles besides leaf senescence and silencing, which could affect flower development, which is important for yield.

The tissue specific expression analysis of *CmNAC60* in *Arabidopsis thaliana* showed that GUS activity was detected in the pistil and stamen, but not in other parts of mature flowers (Figure 6). The result above showed that *CmNAC60* may have a high expression in the pistils and stamens of flowers in oriental melon. The parts of the male and female flowers of oriental melon need to be dissected in order to evaluate the relative expression of *CmNAC60* in these tissues.

### 4.4. CmNAC60 Is a Member of the Melon NAC Transcription Factors

Most transcription factors are localized in the cell nuclei and have transactivation activities. In this study, it was demonstrated that the CmNAC60 protein was localized in the cell nucleus (Figure 4), and the C-terminal domain of CmNAC60 had high transactivation activities in yeast (Figure 5). These results suggest that CmNAC60 is a nucleus-localized NAC transcription factor with a C-terminal transactivation domain

### 4.5. CmNAC60 Overexpression Can Accelerate Leaf Senescence in Arabidopsis

To further determine the function of CmNAC60 in the leaf senescence, transgenic *Arabidopsis* plants overexpressing *CmNAC60* were generated. After dark treatment for five days, the detached leaves of two transgenic lines turned yellow faster than WT. Furthermore, the *CmNAC60* overexpression lines of *Arabidopsis* showed precocious senescence compared with WT after five-weeks of growth under normal conditions. The overexpression line OE-2 had a higher *CmNAC60* expression than OE-8, resulting in more significant early senescence phenotypes of OE-2 in both dark-induced senescence (Figure 7) and nature senescence (Figure 8). The correlation between the level of *CmNAC60* expression and the early senescence phenotypes supported the role of CmNAC60 in promoting leaf senescence in the transgenic *Arabidopsis* plants.

Other than early senescence, we did not observe any other abnormity in the development of the *CmNAC60* overexpressing lines. We did observe the early maturation of *Arabidopsis* siliques in the overexpression line, which is similar to AtNAP [27]. The relative expression level of *CmNAC60* was the highest in the male flowers of oriental melon, and the GUS activity was detected in the pistil and stamen of mature flowers of *Arabidopsis thaliana*, but no obvious difference in the flowers of the transgenic lines overexpressing *CmNAC60* and WT was observed.

Although the overexpression of *CmNAC60* in *Arabidopsis* led to accelerated leaf senescence in the study, more experiments are needed in order to determine the function of CmNAC60 in oriental melon.

## 5. Conclusions

In the study, we identified CmNAC60 as a potential leaf senescence regulator in oriental melon. The expression of *CmNAC60* was significantly higher in the senescing leaves than in the non-senescing leaves. In addition, CmNAC60 was found to be a nucleus-localized NAC transcription factor with a C-terminal transactivation domain. The *CmNAC60* overexpression lines of *Arabidopsis* showed precocious senescence compared with the WT. Collectively, our results showed that *CmNAC60* was associated with leaf senescence and could be a candidate gene for improving the melon yield or extending the postharvest shelf life by delaying leaf senescence in molecular breeding.

## Supplementary Materials

The following are available online at https://www.mdpi.com/2073-4425/10/8/584/s1, Figure S1: Alignment of the nucleotide sequences of *CmNAC60* isolated from oriental melon (*Cucumis melo* var. *makuwa* Makino) and *GeCmNAC60* from the melon genome database, Table S1: Primer sequences used in the study, Table S2: Putative *cis*-acting regulatory elements identified in the *CmNAC60* promoter, Table S3: Putative *cis*-acting regulatory elements identified in the *AtNAP* promoter.
